# The Influence of Water Extraction Methods on the Isolation of Polyphenols and Tannins from Various Ericaceae and Rosaceae Species

**DOI:** 10.3390/plants15050808

**Published:** 2026-03-06

**Authors:** Kristina Ložienė, Evelina Petraitytė

**Affiliations:** 1 Laboratory of Economic Botany, State Scientific Research Institute Nature Research Centre, Akademijos St. 2, LT-08412 Vilnius, Lithuania; 2 Pharmacy and Pharmacology Center, Institute of Biomedical Sciences, Faculty of Medicine, Vilnius University, Geležinio Vilko St. 29A, LT-01112 Vilnius, Lithuania; ev.petraityte@gmail.com

**Keywords:** polyphenols, tannins, water extraction, hot water extraction, maceration, ultrasonic extraction, Rosaceae, Ericaceae, leaves

## Abstract

Most polyphenols (and tannins in their composition), secondary plant metabolites with positive effects on the human body, are soluble in water, which makes them environmentally friendly and the most accessible solvent in everyday life. The aim of this study was to examine the effects of water extraction methods, hot water extraction and maceration, on the amounts of these compounds isolated from plants, compared with ultrasonic extraction, which is not readily available. Seven Ericaceae and four Rosaceae species were selected for study, whose leaves are used in folk and/or official medicine to make herbal teas. Total polyphenolics were determined by the Folin–Ciocalteu method spectrophotometrically and total tannins by calculating the difference between the total and remaining polyphenolic content after tannin precipitation. The results demonstrated that ultrasound was not the most effective method for extracting polyphenols: it yielded the highest polyphenol amounts only from two Rosaceae species, *Potentilla anserina* and *Alchemilla vulgaris*. The hot water extraction of polyphenols was more effective than maceration. Hot water was more effective in extracting polyphenols from evergreen plants. Regardless of the extraction method, most of the polyphenols were extracted with water from *Arctostaphylos uva-ursi* and tannins from *Rhododendron tomentosum* leaves. The studied Ericaceae species accumulate higher-polarity tannins than the studied Rosaceae representatives.

## 1. Introduction

Polyphenols are plant secondary metabolites, which participate in plant defence mechanisms in response to ecological and physiological pressures, such as pathogen and insect attack, UV radiation, and wounding [1]. These compounds, with their broad bioactivity profile, also have positive effects on the human body, especially in the prevention of diseases associated with human ageing, as their main mechanisms of action are related to their antioxidant and anti-inflammatory activity [2,3,4,5,6,7]. Tannins, a highly heterogeneous group of naturally occurring polyphenols in plants, have antimicrobial properties, in addition to their antioxidant, anticancer and other properties; as a result, with the emergence of more and more antibiotic-resistant bacterial species, it is believed that tannins, acting synergistically with antibacterial drugs, could increase the sensitivity of bacteria to antibiotics [8,9,10,11]. People receive polyphenols (including tannins) through their daily diet if they consume enough berries, fruits, and vegetables; however, large amounts of these compounds accumulate in plant leaves, which are not usually used in daily nutrition. Many species rich in polyphenols and tannins in their leaves belong to the Ericaceae and Rosaceae families; some species of these families are used in pharmacy and medicine [12,13]. For example, high amounts of tannins are accumulated in the leaves of *Arctostaphyllos uva-ursi* (Ericaceae) [14]; additionally, the amounts of these compounds, as a minimum requirement for the chemical composition of plant raw material, are specified for the herba of Rosaceae species *Agrimonia eupatoria* and *Alchemilla vulgaris* (min 2.0% and 6.0% tannins, respectively) included in the *European Pharmacopoeia* [13]. Although various natural-origin supplements are produced, teas are among the oldest yet most accessible and fastest ways for the human body to obtain the bioactive compounds accumulated in such plants for both therapeutic and preventive purposes.

Numerous extraction methods and various solvents are used to extract polyphenols and tannins from plants [15,16,17,18]. Often, the organic solvents used in the extraction of biologically active compounds are toxic and environmentally unfriendly. Additionally, most extraction methods are expensive because they require specialised equipment that is typically used in industrial or laboratory settings. However, some polyphenols, as well as most tannins, are soluble in water, which is environmentally friendly, inexpensive, and readily accessible in everyday life (i.e., at home), solvent. Extraction methods commonly used in everyday household life are hot water extraction (pouring boiling water over the medicinal plant material) and maceration (leaving the plant material in water for a while until it is fully soaked) [15].

Therefore, the aim of this work was to test how these two water extraction methods affect the total polyphenols and total tannins extracted from plant leaves and to compare them with the more modern, recently popular ultrasonic extraction, which is used in industry and laboratories but is not available in everyday life. Various species from the Ericaceae and Rosaceae families were selected for this study (some species were deliberately chosen because these compounds had been poorly studied in them) to compare the efficiencies of different water extraction methods across species and families.

## 2. Results

### 2.1. Comparison of Total Polyphenols in Ericaceae and Rosaceae Species Using Different Water Extraction Methods

**Figure 1:**
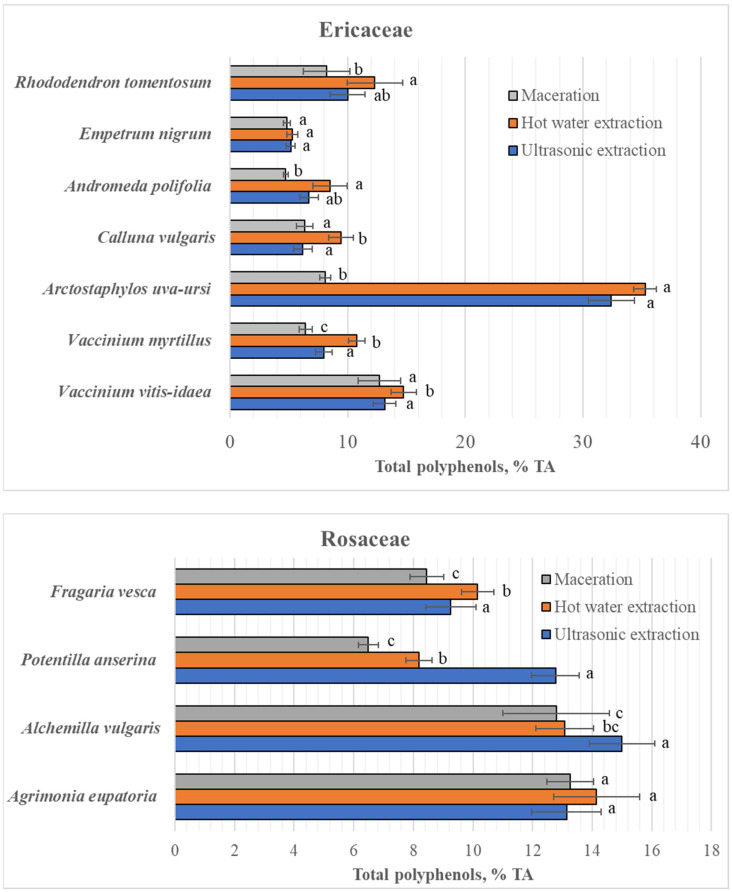
Contents of total polyphenols (mean ± standard deviation) in leaves of different Ericaceae and Rosaceae species, using different water extraction methods. Different and same letters above columns denote significant and non-significant differences, respectively, between water extraction methods within species (selected significance level: *p* < 0.05); TA—tannin acid.

The total polyphenols extracted by different water extraction methods in the studied species varied within wide ranges: from 4.7 ± 0.2% (in *A. polifolia* after maceration) to 35.3 ± 1.0% (in *A. uva-ursi* after hot water extraction) (Figure 1).

The highest content of total polyphenols in all studied Ericaceae species was determined by hot water extraction, and the content of these compounds extracted by this method in the leaves of *V. vitis-idaea* and *V. myrtillus* as well as in a mixture of leaves and flowers of *C. vulgaris* statistically significantly (*p* < 0.05) differed from the content of these compounds extracted using the other two water extraction methods (Figure 1). Using ultrasonic extraction, a lower total polyphenol content was extracted, but only in *C. vulgaris*, *V. myrtillus* and *V. vitis-idaea* did their content statistically significantly (*p* < 0.05) differ from the content extracted with hot water. Maceration with room-temperature water in the dark for 24 h resulted in the lower total polyphenol content extracted from the absolute majority of species of this family, which were statistically significantly (*p* < 0.05) different (except for *E. nigrum* leaves) from the total polyphenols determined by extraction with hot water. A particularly large difference in the content of extracted polyphenolic compounds was observed for *A. uva-ursi*, with the maceration of *A. uva-ursi* leaves yielding 4.4 times fewer polyphenolic compounds than hot water extraction. Meanwhile, from the leaves of other Ericaceae species, maceration extracted 9% to 44% lower total polyphenol content (depending on the species) than hot water extraction. The content of polyphenolic compounds extracted by ultrasonic extraction was very similar and did not differ significantly from that extracted by maceration, except in the cases of *A. uva-ursi* and *V. myrtillus*, where the content of total polyphenols extracted by ultrasonic extraction differed significantly (*p* < 0.05) from that extracted by maceration. When extracting polyphenolic compounds from *E. nigrum* leaves, the extraction method did not significantly affect total polyphenolic content: the values obtained with all tested methods did not differ significantly.

Unlike in Ericaceae species, not all of the studied Rosaceae species yielded the highest content of polyphenolic compounds extracted using hot water extraction: compared to other water extraction methods, the highest content of polyphenolic compounds was extracted using this method only from *F. vesca* (10.2 ± 0.6%) and *A. eupatoria* (14.2 ± 1.5%) leaves, and only in the case of *F. vesca* did their content differ statistically significantly (*p* < 0.05) from the amounts extracted by other water extraction methods (Figure 1). The most polyphenolic compounds from the leaves of two other Rosaceae species were extracted using ultrasonic extraction: the content of polyphenols extracted by ultrasonic extraction from *A. vulgaris* and *P. anserina* was statistically significantly different from the content of polyphenols extracted by maceration or hot water extraction. The lowest total polyphenolic content in Rosaceae species was also obtained by macerating with distilled water at room temperature for 24 h in the dark. Only in the case of *A. eupatoria* did the contents of total polyphenols extracted by all three water extraction methods not differ significantly.

**Figure 2:**
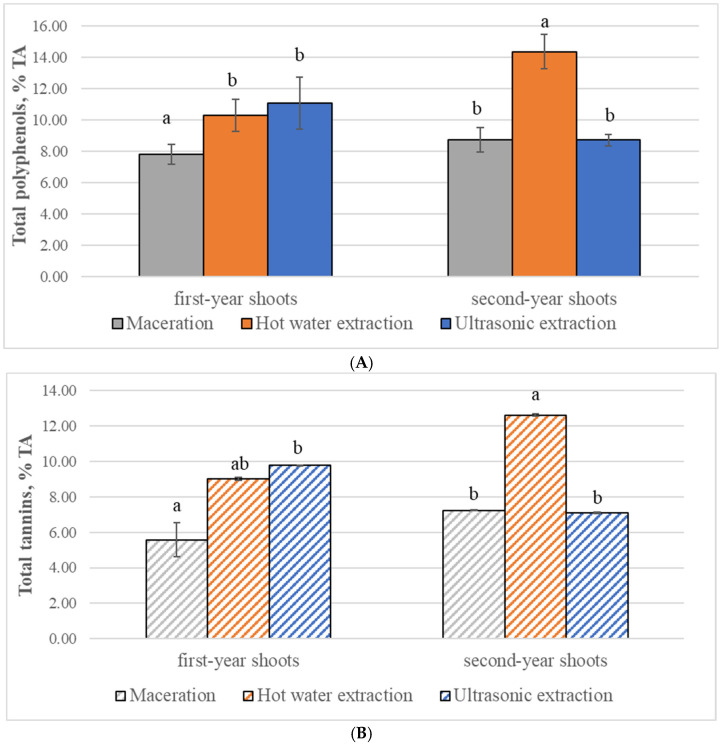
Contents of total polyphenols (mean ± standard deviation) (**A**) and total tannins (**B**) in mix of leaves and flowers of *Rhododendron tomentosum* (Ericaceae) using different water extraction methods. Different and same letters above columns denote significant and non-significant differences, respectively, between extraction methods in first-year and second-year shoots (selected significance level: *p* < 0.05); TA—tannin acid.

Interestingly, although the highest content of polyphenols was extracted from *R. tomentosum* using hot water extraction, different results were obtained when the leaves of first-year shoots and those of second-year shoots were examined separately (Figure 2A). 

Hot water extraction was also the most effective water extraction method for extracting polyphenols from the leaves of second-year shoots; meanwhile, the highest content of polyphenols from the leaves of first-year shoots was extracted using ultrasonic extraction, although the content of these compounds extracted by this method did not differ significantly from their content extracted with hot water extraction. 

### 2.2. Comparison of Total Tannins in Ericaceae and Rosaceae Species Using Different Water Extraction Methods

**Figure 3:**
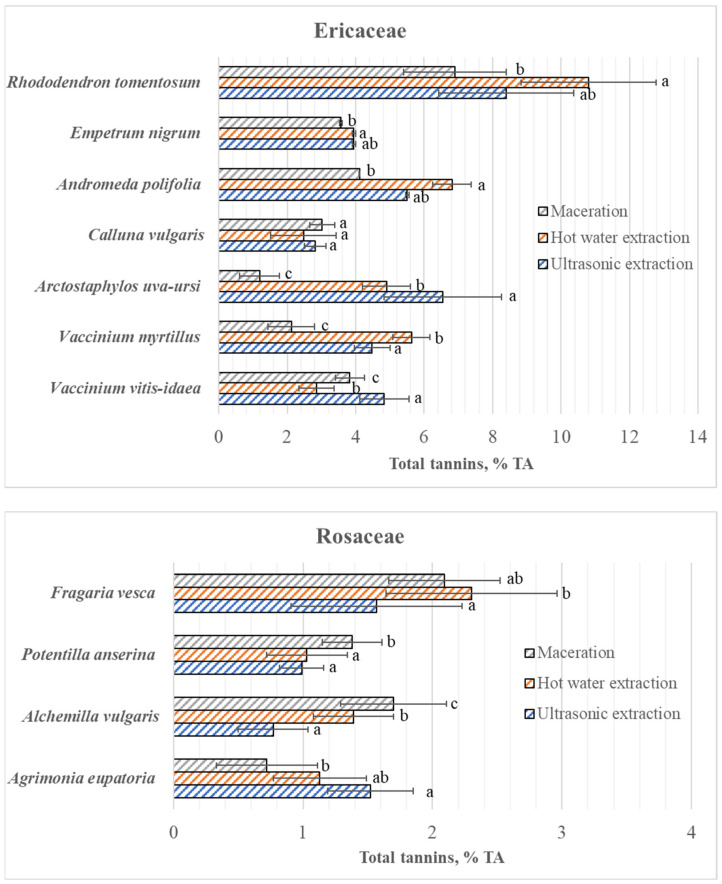
Contents of total tannins (mean ± standard deviation) in leaves of different Ericaceae and Rosaceae species, using different water extraction methods. Different and same letters above columns denote significant and non-significant differences, respectively, between water extraction methods within species (selected significance level: *p* < 0.05); TA—tannin acid.

The content of total tannins extracted from the studied species by different water extraction methods varied from 1.2 ± 0.6% (in *A. uva-ursi* after maceration) to 10.8 ± 2.0% (in *R. tomentosum* after hot water extraction) (Figure 3). The results presented in Figure 3 show that the plants of the Ericaceae family species accumulate more tannins than the plants of the studied Rosaceae species. Also, in the studied Rosaceae species, tannins constituted a significantly lower percentage of the total polyphenols than in the Ericaceae species: in Rosaceae and Ericaceae, depending on the studied species and the extraction method used, tannins constituted from 5% to 25% and from 14% to 88% of the total polyphenols, respectively (Table 1).

Unlike polyphenols, there was no single effective water extraction method for tannins in Ericaceae species (Figure 3). For example, the ultrasonic extraction method was the most effective for tannin extraction from *V. vitis-idaea* and *A. uva-ursi* leaves, where the total tannin content extracted by this method significantly (*p* < 0.05) differed from the tannin content extracted by other methods. Using hot water extraction, which was the most effective method for extracting polyphenols from all investigated Ericaceae species, most tannins were extracted from *V. myrtillus*, *R. tomentosum* and *A. polifolia* leaves, of which only in the case of *V. myrtillus* was the content significantly (*p* < 0.05) higher than that extracted by the other two methods. Meanwhile, the content of tannins extracted from *C. vulgaris* and *V. vitis-idaea* by hot water was the lowest; in the case of *V. vitis-idaea*, it was significantly (*p* < 0.05) different, and in the case of *C. vulgaris*, it was not different from the tannin contents extracted by the other methods. As with the extraction of polyphenols, maceration with room-temperature water in the dark for 24 h resulted in the lowest content of total tannins in the absolute majority of species of this family (except *C. vulgaris* and *V. vitis-idaea*), but only in the case of *A. uva-ursi* and *V. myrtillus* did their contents significantly (*p* < 0.05) differ from the content determined by other extraction methods. In the case of the Rosaceae family, only *F. vesca* leaves (using maceration and boiled water) yielded more than 2% of tannins (Figure 3). And only in the case of *F. vesca* was the same extraction method (i.e., hot water extraction) the most effective for extracting both tannins and polyphenols. The highest tannin content (which was statistically significant (*p* < 0.05)) from *P. anserina* and *A. vulgaris* leaves was extracted using maceration. Ultrasonic extraction yielded the highest amount of tannins only from *A. eupatoria* leaves; maceration was the least effective for this raw material; and the content of total tannins extracted from *A. eupatoria* leaves by this method was half that of the content extracted by ultrasonic extraction.

The highest tannin content was isolated from *R. tomentosum* by hot water extraction (Figure 3); however, different results were obtained when first- and second-year shoots were examined separately (Figure 2B). As in the case of polyphenols, the highest total amount of tannins was isolated from the leaves of the first- and second-year shoots by ultrasonic and hot water extraction, respectively.

When extracting tannins from *R. tomentosum* leaves of different ages, the distribution of extraction efficiency was very similar to the distribution of total polyphenol extraction efficiency (Figure 2). However, unlike in the case of polyphenols, the tannin content extracted from the leaves of first-year shoots by maceration did not differ significantly from the tannin content extracted by hot water.

## 3. Discussion

The main pharmacologically active compounds of *A. uva-ursi*, due to which it is used in official medicine, are simple polyphenols, such as hydroquinone-β-D-glucopyranoside (arbutin) [19], as well as methylarbutin and free aglycones, which do not belong to tannins [12]. Due to the high arbutin content, raw material from *A. uva-ursi* leaves (*Uvae ursi folium*) is included in the *European Pharmacopoeia* [13]. Therefore, although polyphenols were extracted from *A. uva-ursi* leaves in the highest amount (regardless of the water extraction method used, the average polyphenol content in the leaves of these plants was 25.3 ± 14.0%); compared to the other Ericaceae species studied, tannins in *A. uva-ursi* leaves constituted the lowest proportion of polyphenols (14–20%). Meanwhile, as indicated in the literature, the leaves of the evergreen shrub *R. tomentosum* are rich in polyphenols, one of the most abundant of which is (+)-catechin, which is classified as a tannin [20,21]. Therefore, it is not for nothing that tannins were extracted from *R. tomentosum* in the largest amount, and depending on the age of the leaves and the extraction method, their share in the total amount of polyphenols ranged from 72 to 88%. Based on the established higher percentages of tannins in the total amount of polyphenols, the studied Ericaceae species should accumulate more tannins of higher polarity, those with more hydroxyl groups in their molecular structure and those that are better soluble in polar solvents (for example, water) than the studied Rosaceae representatives. However, it should be acknowledged that, despite its widespread use, the Folin–Ciocalteu assay is not fully specific to phenolic compounds: the Folin–Ciocalteu reagent can also react with other reducing substances present in plant extracts, such as ascorbic acid, reducing sugars, and certain amino acids, which may lead to a partial overestimation of total polyphenol content [22,23,24]. While this limitation does not affect the comparative evaluation of extraction efficiency within a species, interspecific comparisons should be interpreted with caution, as the obtained values may reflect overall reducing capacity rather than the exact phenolic content.

Many extraction methods incorporating different technologies (such as ultrasound, microwave, high pressure, high voltage, and mechanical forces) as well as different organic and inorganic solvents are used to extract biologically active compounds from medicinal plant materials [1,15,25]. Water as an inorganic solvent may be less effective than organic solvents if the compounds accumulating in plants are more soluble in low-polarity solvents [15]. However, water is a low-cost, non-toxic, and environmentally friendly solvent; it is readily available in everyday life, and polyphenols (including tannins, which constitute a large group of polyphenolic compounds) are water-soluble [26]. The efficiency of extraction can be influenced by various extraction parameters, such as extraction time, temperature, solvent pH, etc. [1,10]. In our case, focusing on methods for producing aqueous extracts of medicinal plants commonly used in everyday life, we chose water extraction methods that differ in extraction temperature: traditionally, pouring boiling water over the plant material or keeping it in room-temperature water for a long time (maceration). It is known that increasing the temperature reduces the solvent’s surface tension and viscosity, allowing it to better penetrate the extractable material and moisten it; higher temperatures also improve the diffusion and solubility of chemical compounds, making cell walls more permeable [27]. However, it is not entirely clear how high temperatures affect the stability of polyphenolic compounds. For example, studies demonstrated that at high temperatures, plant polyphenols can degrade or undergo undesirable reactions such as enzymatic oxidation [28,29]; meanwhile, pressurised liquid extraction studies have shown high polyphenolic yield at temperatures well above even 100 °C, and an increase in extraction temperature up to 180–200 °C correlates to an increase in polyphenol content [30]. Our results showed that maceration was less effective in extracting polyphenols than hot water extraction for the studied plants, especially for representatives of the Ericaceae family: the maceration of Ericaceae species, depending on the species, extracted 9–77% (average 35.9 ± 22.4%) and from Rosaceae species, only 2–21% (average 11.5 ± 9.0%) less polyphenols than the same species when hot water extraction was applied. A particularly large advantage of hot water extraction over maceration was observed in *A. uva-ursi*. This suggests that high water temperature facilitates the extraction of polyphenolic compounds and that they are resistant to boiling water (at least for a short exposure). Hot water extraction, a more efficient method of polyphenol extraction than maceration, has also been reported to yield aqueous extracts from powdered leaves of *Ribes nigrum* and *Crysanthellum americanum* [31]. 

Higher temperatures during water extraction help break down plant cell walls, facilitating the extraction of polyphenols contained within cells and/or cell walls into the solvent [32]. The investigated Ericaceae species (except *V. myrtillus*) are evergreen plants. The literature indicates that evergreen (unlike deciduous plants), to protect chloroplasts and facilitate CO_2_ diffusion in cold and dry winters, often have leaves with thicker cell walls, waxy cuticles and robust internal structures (like thicker mesophyll) [33]. Therefore, the mechanical disruption of such cells will be more difficult. This may be another reason why maceration with room-temperature water was less effective for the investigated Ericaceae species, and the amounts of polyphenols extracted by maceration in all studied species of this family were statistically significantly lower than those extracted with hot water. Meanwhile, all studied Rosaceae species are not evergreen; therefore, according to the above-mentioned literature source, their leaf cell walls should be thinner and, probably, more easily degraded. This may account for the smaller differences observed between extraction methods in the studied Rosaceae species.

Ultrasonic extraction, although inaccessible for everyday human use, has recently been often reported as a method for obtaining a higher yield of bioactive compounds, consuming less time, energy and solvents compared to conventional solid–liquid extraction methods, and due to its ease of use and it not requiring expensive equipment, it is widely used in industry and laboratories [25,34]. Ultrasound severely ruptures plant cell walls, facilitating the release and diffusion of their components [35,36]. Therefore, theoretically, there was reason to believe that ultrasonic extraction would be more effective for Ericaceae plants, which have thicker cell walls. However, our results demonstrated that this extraction method did not yield the highest polyphenol content in any of the studied Ericaceae species. This may be related to the concentrated diffuse energy generated by ultrasonic cavitation during ultrasonic vibration, which can cause various side effects, such as extremely high temperatures and pressures, shock waves, microjets, and the formation of hydroxyl radicals from water molecules, which often degrade bioactive compounds in solution [25].

The solubility of tannins in water may depend on the integration of tannins into plant cell walls or organoid membranes, when, after the ester bonds are broken, tannins bind to other molecules and form complexes with proteins and polysaccharides, and this may also depend on the degree of polymerisation of tannins (hydrolysable tannins are more water-soluble that more polymerised condensed tannins also known as proanthocyanidins) [37,38,39]. Tannins in plant cells are polymerised and form condensed tannins, which are stored in tannosomes, chloroplast-derived organelles; in tannosomes, they do not interfere with plant metabolism and do not interact with proteins [40]. If ultrasound can disrupt cell walls, aiding the release and diffusion of components from them [35,36], it should also disrupt the membranes of tannosomes, and components accumulated in these organelles could be leached into the solvent. As a result, it can be assumed that more highly polymerised condensed tannins can accumulate in the leaves of *A. uva-ursi* and *V. vitis-idaea*, as higher tannin levels were observed following ultrasonic extraction compared with hot water extraction. The literature also indicates that *A. uva-ursi* leaves accumulate not only hydrolysable gallotannins but also condensed tannins [37,41], and in *V. vitis-idaea* leaves, proanthocyanidins represent up to 71% of the total polyphenols [42]. 

In contrast to the Ericaceae studied, in species of the Rosaceae family, the total tannin percentages in total polyphenolic amounts were much lower, the maceration for tannin extraction was more effective (especially from *P. anserina* and *A. vulgaris*), and ultrasonic extraction did not increase tannin yield. As mentioned above, this could be related to anatomical differences in leaf cells (such as cell wall and cuticle thickness) characteristic of these plant families. Interestingly, using ultrasound with methanol as the solvent, the total tannin content in *P. anserina* leaves studied in Poland was 0.81–0.91% [43], i.e., very similar to that in our study using ultrasonic extraction with water as the solvent. Therefore, despite the effect of different growth conditions and the possible chemical polymorphism of *P. anserina*, it can be assumed that water is no worse a solvent than methanol for extracting tannins from plants of this species, and it has a greater advantage because it is a green solvent. The exception of *A. eupatoria* could be explained by the fact that, as indicated in the literature, this species accumulates mainly proanthocyanidins and only a small amount of ellagitannins [8]. As mentioned above, condensed tannins (proanthocyanidins) accumulate in tannosomes, and ultrasound can help disrupt the membranes of these organelles and leach the condensed tannins into the solvent.

## 4. Materials and Methods

### 4.1. Plant Material

The raw materials of seven species of the Ericaceae family (*Vaccinium vitis-idaea*, *Vaccinium myrtillus*, *Arctostaphylos uva-ursi*, *Calluna vulgaris*, *Andromeda polifolia*, *Empetrum nigrum*, and *Rhododendron tomentosum*) and 4 species of the Rosaceae family (*Agrimonia eupatoria*, *Alchemilla vulgaris*, *Fragaria vesca*, and *Potentilla anserina*) were collected separately from natural habitats in Lithuania; only leaves were collected as raw material, except *C. vulgaris*, for which a mixture of leaves and flowers was collected. *A. uva-ursi* and *C. vulgaris* plant raw materials were collected during fruit ripening and flowering, respectively, in a forest habitat in Valkininkai (Varėna district municipality); *V. vitis-idaea*, *V. myrtillus* and *F. vesca* leaves were collected during flowering in a forest habitat in Braziūkai (Kazlų Rūda municipality); *A. vulgaris* and *A. eupatoria* leaves were collected during flowering in a meadow in Verkiai (Vilnius); *P. anserina* leaves were collected during flowering in a meadow in Vilkaviškis (Marijampolė county); leaves of *R. tomentosum* (first-year and second-year separately), *E. nigrum* and *A. polifolia* were collected during fruit ripening in a swamp Raudonoji bala (Vilnius district). All collected plants were dried separately at room temperature in a closed, well-ventilated room, away from direct sunlight. 

### 4.2. Preparation of Extracts

The air-dried raw material of each plant species was ground separately using a electric grinder mill GM 200 (Retsch, Germany) and sieved through a sieve with 0.5 mm holes. For the preparation of each extract, 400 mg of ground raw material was weighed. Extraction with water was performed in three different ways: (1) hot water extraction—the plant material was poured with 20 mL of boiling water and maintained for 20 min; (2) maceration—the plant material was poured with 20 mL of room-temperature water and kept in the dark for 24 h; (3) ultrasonic extraction—the plant material was poured with 20 mL of room-temperature water and kept in a Bandelin Sonorex ultrasonic bath (Bandelin, Germany) for 20 min at room temperature. After that, the extracts were centrifuged for 10 min at 3000× *g* at +4 °C in an Eppendorf 5430R centrifuge (Eppendorf, Germany). Such tannin-containing extracts of each type for each raw material were prepared in triplicate.

### 4.3. Determination of Total Polyphenol Content

The total polyphenolic content was determined spectrophotometrically using the Folin–Ciocalteu method [44]; a Biochrom Libra S32 PC spectrophotometer (Biochrom, UK) was used for the analysis. The obtained tannins-containing extracts were diluted in a ratio of 1:5 or 1:10. Subsequently, 0.02 mL of the diluted extract was poured into a graduated test tube, making the volume 0.5 mL with distilled water, and 0.25 mL of Folin–Ciocalteu and 1.25 mL of 20% sodium carbonate solution were added; the tube was vortexed, and after 40 min, the optical density was measured at a wavelength of 725 nm using 1 cm diameter cuvettes. The content of total polyphenolics was calculated as tannic acid equivalents from the calibration curve and expressed as a percentage in an absolutely dry raw material. Each diluted tannin-containing extract was examined in 3 replicates.

### 4.4. Determination of Total Tannin Content 

To determine the total tannins, polyvinylpyrrolidone (PVPP) powder was used, which binds tannins and removes them from tannin-containing extracts [44]. A total of 0.1 g of PVPP was added to centrifuge tubes; 1 mL of distilled water and 1 mL of diluted tannin-containing extract were added. The mixture was shaken and incubated for 15 min at +4 °C, then centrifuged for 10 min at 3000× *g* at +4 °C. The total polyphenols remaining in the resulting supernatants were measured spectrophotometrically using the Folin–Ciocalteu reagent and calculated as described in Section 4.3. The total tannins were calculated by subtracting the total polyphenols in the supernatant from the total polyphenols determined in the tannin-containing extract. The total tannin content was determined in 3 replicates.

### 4.5. Determination of Absolute Dry Matter Content in Plant Raw Materials

The absolute dry matter content was expressed as a percentage and calculated by subtracting the determined moisture content from the air-dried raw material. The moisture content of the air-dried raw material was determined by thermal drying: 2 g of crushed air-dried raw material was heated for 4 h at 104 °C in a Memmert UN55 (Memmert GmbH, Germany) heating oven; the air-dried raw material of each species was heated in three replicates.

### 4.6. Statistical Analysis

Statistical data processing was carried out using STATISTICA^®^ 10 and MS Excel 2016. Means and standard deviations were calculated, and graphs were drawn with MS Excel 2016. The Kruskal–Wallis test was used to analyse differences in the contents of total polyphenols and total tannins extracted using different water extraction methods within each species; the analysis was conducted in STATISTICA^®^ 10.

## 5. Conclusions

Although most polyphenols and tannins are highly soluble in water, which makes them environmentally friendly and the most accessible solvent in everyday life, not all investigated water extraction methods—hot water extraction, maceration at room temperature, and ultrasonic/extraction—were equally effective for the extraction of these biologically active secondary plant metabolites from the leaves of Ericaceae and Rosaceae plants. From Ericaceae plants, most of which are evergreen with characteristic thicker cell walls, waxy cuticles and robust internal structures, and from some Rosaceae plants, more polyphenols can be extracted with hot water than with room-temperature maceration. However, maceration was the most effective method for extracting tannins from Rosaceae plants. Ultrasonic extraction, which is widely used in industry and laboratories but is not accessible to everyday users, was not very effective at extracting either polyphenols or tannins and performed better than the other two water extraction methods in only a few of the species studied.

## Figures and Tables

**Table 1:** Percentage of extracted total tannins in total amount of polyphenolics using different water extraction methods.

Species		Percentage of Total Tannins in the Total Polyphenols		
		Ultrasonic Extraction	Hot Water Extraction	Maceration
*Vaccinium vitis-idaea*		37	19	30
*Vaccinium myrtillus*		56	52	33
*Arctostaphylos uva-ursi*		20	14	15
*Calluna vulgaris*		46	26	48
*Andromeda polifolia*		52	80	87
*Empetrum nigrum*		77	74	74
*Rhododendron tomentosum*		84	88	84
*Agrimonia eupatoria*		12	8	5
*Alchemilla vulgaris*		5	11	13
*Potentilla anserina*		8	13	21
*Fragaria vesca*		17	23	25
*Rhododendron tomentosum*	first-year shoots	88	88	72
	second-year shoots	81	88	83

## Data Availability

Data are contained within the article.
